# The Evaluation of Damage Effects on MgO Added Concrete with Slag Cement Exposed to Calcium Chloride Deicing Salt

**DOI:** 10.3390/ma11050793

**Published:** 2018-05-14

**Authors:** Jae-Kyeong Jang, Hong-Gi Kim, Jun-Hyeong Kim, Jae-Suk Ryou

**Affiliations:** 1 Department of Civil and Environmental Engineering, Hanyang University, 222, Wangsimni-ro, Seongdong-gu, Seoul 05510, Korea; holyjang915@gmail.com; 2 Concrete Laboratory, Department of Civil and Environmental Engineering, Hanyang University, 222, Wangsimni-ro, Seongdong-gu, Seoul 05510, Korea; dmkg1404@naver.com (H.-G.K.); bspring@nate.com (J.-H.K.)

**Keywords:** MgO, CaCl_2_ deicer, durability, slag cement, SEM-EDAX, XRD, chloride penetration

## Abstract

Concrete systems exposed to deicers are damaged in physical and chemical ways. In mitigating the damage from CaCl_2_ deicers, the usage of ground slag cement and MgO are investigated. Ordinary Portland cement (OPC) and slag cement are used in different proportions as the binding material, and MgO in doses of 0%, 5%, 7%, and 10% are added to the systems. After 28 days of water-curing, the specimens are immersed in 30% CaCl_2_ solution by mass for 180 days. Compressive strength test, carbonation test, chloride penetration test, chloride content test, XRD analysis, and SEM-EDAX analysis are conducted to evaluate the damage effects of the deicing solution. Up to 28 days, plain specimens with increasing MgO show a decrease in compressive strength, an increase in carbonation resistance, and a decrease in chloride penetration resistance, whereas the S30- and S50- specimens show a slight increase in compressive strength, an increase in carbonation resistance, and a slight increase in chloride penetration resistance. After 180 days of immersion in deicing solution, specimens with MgO retain their compressive strength longer and show improved durability. Furthermore, the addition of MgO to concrete systems with slag cement induces the formation of magnesium silicate hydrate (M-S-H) phases.

## 1. Introduction

Concrete used in road structures are often exposed to harmful external factors, one of which is the deicing products widely used to remove ice and snow in cold regions. These deicing chemicals usually consist of chloride-based salts such as sodium chloride (NaCl), calcium chloride (CaCl_2_), and/or magnesium chloride (MgCl_2_). Deicers directly affect the durability and strength of concrete structures in ways both physical and chemical [1]. The physical damage from deicers are manifested in the form of cracking and salt scaling, which may not cause severe damage by itself, but will make the concrete structures more susceptible to frost action or chemical attack. The chemical damage from deicers come from reactions between the deicer ions and the cementitious matrix: leaching of calcium hydroxide (CH), decalcification of calcium silicate hydrate (C-S-H), formation of brucite (Mg(OH)_2_), and the conversion of C-S-H to magnesium silicate hydrate (M-S-H) [2]. Other forms of chemical damage come from the formation of Friedel’s salt (3CaO∙Al_2_O_3_∙CaCl_2_∙10H_2_O), Kuzel’s salt (3CaO∙Al_2_O_3_∙1/2CaSO_4_∙1/2CaCl_2_∙11H_2_O), and/or calcium oxychloride (common type known as the 3:1:12 form—3Ca(OH)_2_∙CaCl_2_∙12H_2_O) which could undergo even without freezing and melting cycles [3,4,5]. Furthermore, deicing salts have been reported to cause corrosion damage to road structures made with reinforced or prestressed concrete. The steel embedded in concrete is protected from corrosion by the alkalinity of the cement matrix, which forms a passive film on the steel surface. The problem arises when these structures are exposed to external sources of chloride ions. In road structures where deicing salts are used, the ingress of chloride ions poses a great threat to the reinforcing steel. The formed protective passive film will undergo localized breakdown when the Cl^−^ content reaches critical content, often referred to as the chloride threshold, and corrosion will occur in the presence of water and oxygen on the steel bars [6,7,8,9].

To prevent damages from deicers and mitigate the detrimental effects of chloride ingress, the usage of supplementary cementing materials (SCM) have long been utilized. SCMs have shown a wide range of application in concrete structures either as replacements for Portland cement (PC) or as additives in the concrete mix. Aside from the clear environmental advantages via the mitigation of anthropogenic carbon emission that will aid in lowering greenhouse gas levels globally [10], using SCMs such as slag cement or fly ash (FA) in the right proportions can induce improved compressive strength and chloride resistance [11]. Slag cement has been proven to modify the microstructure of cementitious paste, reducing the pore sizes and cumulative pore volume. They have shown such characteristics as increased compressive strength at latter ages, decreased chloride ion penetration, increased resistance to sulfate attack, and alkali–silica reaction [12]. The durability of the concrete itself and the improvement of it is an important issue concerning elongated service life of concrete structures. Slag cement has been proven effective in decreasing chloride conductivity due to the additional C-S-H phases and higher alumina content that refine the microstructure [13].

Apart from the usage of SCMs, another field of interest has been towards the broad group of MgO-based cements and the usage of MgO as an expansive agent in concrete. The first reported practical applications have mainly been in China, where MgO concrete was applied to the construction of dams. Over the past three decades, MgO concrete has been successfully applied to nearly 30 dams and have shown shrinkage compensating of concrete in the long term [14]. Since then, many approaches have been developed as a means to utilize MgO in concrete mixes. Such approaches include studies on magnesium carbonate and reactive magnesia cements [14,15], magnesium phosphate cements [16,17], magnesium oxysulfate cements [18,19], and M-S-H cements [20,21]. Of these MgO-based cements, the addition of reactive MgO as an expansive additive has shown great potential in compensating both thermal shrinkage at late age and autogenous shrinkage of concrete at a relatively early age [22]. While MgO cement with high content of periclase has had limitations regarding the unsoundness of cement related to volume stability, reactive MgO can be separately produced by calcining magnesite at 900–1200 °C. This enables the design and control of hydration reactivity and expansion properties by adjusting the calcining conditions [22,23]. Adding to that, the durability characteristics of concrete with FA and reactive MgO have showed improved resistance to carbonation, chloride attack, and sulfate attack in the long term by decreasing 0.03–0.3 μm pores associated with strength and ion diffusion [24].

Although much research on the topics of SCMs and MgO have been conducted respectively, the possibilities of incorporating MgO to SCMs for pavement systems have been limited. Previous researches on damages from deicers have comprehensively studied the effects of calcium chloride deicing salt on the concrete system in ways both mechanical and chemical. Past research regarding the damage of calcium chloride deicing salt on high-strength concrete specimens have shown that calcium chloride can be severely detrimental to concrete systems. The reaction between the deicer and the cement matrix induces formations of expansive salts, and the combined damage from chemical reactions with physical damages from freeze–thaw cycles induces further damage [25]. Another research that studied the effects of deicer exposure using field emission scanning electron microscopy and energy-dispersive X-ray spectroscopy showed the damage mechanisms of calcium chloride deicer with inhibitors [26]. This research focused on the chemical damages of deicers on concrete systems by showing surfaces exposed to CaCl_2_: the formation of calcium-rich type II C-S-H phases, hydrated calcium oxychloride, and hydrated magnesium oxychloride were identified [26,27,28,29]. Furthermore, another research on the behavior of cement matrix exposed to CaCl_2_ at room temperature has shown that calcium oxychloride will form without thermal cycling, which in turn be destructive and cause damages and cracks [3].

As mentioned in the previous paragraph, research on concrete systems incorporating slag cement and MgO and the damage to these systems triggered by CaCl_2_ deicers have not been conducted. The objective of this research is to incorporate slag cement and reactive MgO, and then evaluate the consequent damage effects triggered by CaCl_2_ deicer solution. In assessing the damages to different mix proportions of concrete incorporating slag cement and MgO, three categories of concrete mixes with different binder composition were prepared: ordinary Portland cement (OPC) without SCMs as a control group, 30% replacement of OPC by slag cement, and 50% replacement of OPC by slag cement. These three categories each included four different dosages of MgO respectively: 0%, 5%, 7%, and 10% addition of MgO relative to the weight of the binder. A total of 12 concrete mixes were water-cured for 28 days and then immersed in deicer solution that was 30% CaCl_2_ by mass for up to 180 days. To analyze the damages to the concrete specimens, compressive strength test, carbonation test, chloride penetration test, chloride content test, XRD, and SEM-EDAX were conducted.

## 2. Materials and Methods 

### 2.1. Materials

In this study, Type I PC in accordance with KS L 5201, manufactured in Korea, was used as the main binder and slag cement was used as cement replacement. The MgO additive used in this research was lightly burnt and calcined at approximately between 850–1200 °C (as opposed to dead burnt MgO which is calcined at between 1500–1800 °C). The chemical composition and physical properties of the binders and the MgO are presented on Table 1.

The coarse aggregates used in this study were crushed granite aggregates from Gyeonggi-do, South Korea. The fine aggregates were also from Gyeonggi-do. Table 2 shows the physical properties of the aggregates. A polycarboxylic based superplasticizer was added to improve workability and control slump. The deicer used in this study was CaCl_2_ and the concentration of the CaCl_2_ solution was 30% by mass.

### 2.2. Specimen Preparation

Concrete cylinder specimens (ø100 × 200 mm) were prepared according to KS F 2403. The mix proportions of the concrete specimens are shown in Table 3. The water to binder ratio was set at 42.5%, and the sand to aggregate ratio was 39.2% for all specimens. Upon preliminary experiments using mortar, the replacement levels of OPC with slag cement were set at 30% and 50%. MgO dosages were limited at 10% due to the specimens dropping in compressive strength.

**Figure 1:**
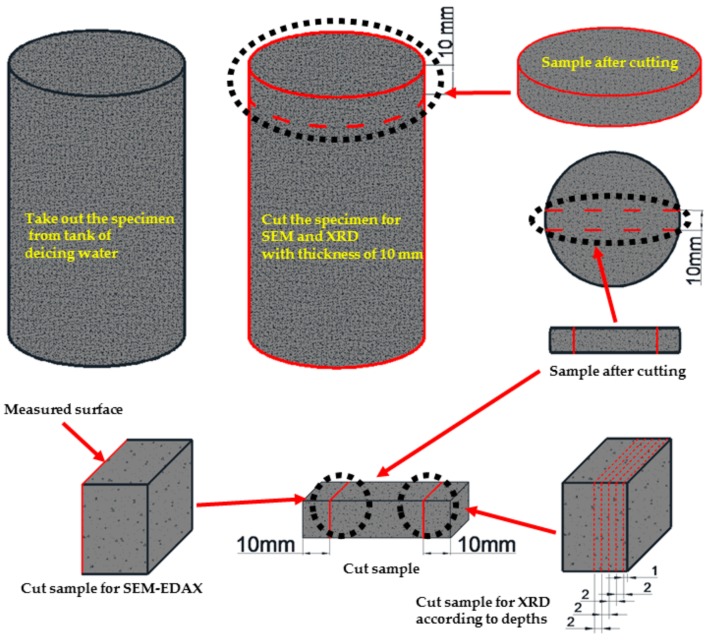
Specimen preparation for XRD and SEM-EDAX analyses.

The specimens were demolded after 24 h and subsequently immersed in water for curing at 20 ± 2 °C. The cylinder specimens were used as they were in compressive strength tests. For XRD and SEM-EDAX analyses, the upper parts of the specimens were cut into 10 mm thick discs. The first disc from the apex was discarded due to the top surface being in direct contact with the solutions to which the specimens were exposed. The second discs were used for XRD and SEM-EDAX analysis. From the 10 mm thick disc, the center strip was cut perpendicularly into a disc with a diamond-crusted blade that was cooled with deionized water. Then, the end parts of the strip near the surface were used for analysis. One millimeter of the samples was then ground from the lateral surface inward for XRD analysis to give results for different depths into the sample’s principal axis as illustrated in Figure 1.

### 2.3. Experimental Methods

#### 2.3.1. Compressive Strength

Compressive strength test was conducted to all the water-cured specimens after 3, 7, and 28 days. After all the specimens were water-cured, the specimens were immersed in deicing solutions. The compressive strengths of these specimens were analyzed at 30, 60, 120, and 180 days after immersion (58, 88, 148, and 208 days after mixing). A universal testing machine was used to conduct the compressive strength tests in accordance with KS F 2405. Each compressive strength value was obtained by testing three specimens and the average value was used.

#### 2.3.2. Accelerated Carbonation

The accelerated carbonation test was conducted according to KS F 2403 in order to analyze the degree of carbonation of the specimens after 28 days of water-curing. Cylinder specimens were cut into smaller cylinders (ø100 × 50 mm) and then all surfaces except for the top where carbonation depth was to be measured were coated to prevent contact to external CO_2_. Then the specimens were placed in a chamber with 5% CO_2_ concentration, 20 ± 1 °C, and 60% RH. The specimens were kept in the chamber for 28 days and phenolphthalein was used to measure the depth of neutralization on the specimens after they were split in half vertically.

#### 2.3.3. Chloride Penetration Test

The accelerated chloride penetration test was conducted according to NT Build 492 [30] to analyze the chloride penetration resistance of the specimens after 28 days of water-curing. The specimens were prepared by cutting 50 ± 2 mm thick slice from the central portion of the cylinder. The end surface that was nearer to the as-cast surface was the one to be exposed to the chloride solution (catholyte). After applying external voltage, the chloride penetration depth was measured by using AgNO_3_ solution. With the measured penetration depth, the nonsteady-state migration coefficient was calculated by Equation (1):

(1) $$D_{nssm}=\frac{0.0239\left(273+T\right)L}{\left(U-2\right)t}\;\left(x_{d}-0.0238\sqrt{\frac{\left(273+T\right)Lx_{d}}{U-2}}\right),$$

where *D_nssm_* is the nonsteady-state migration coefficient (×10^−12^ m^2^/s), *U* the absolute value of the applied voltage (V), *T* the average value of the initial and final temperatures in the anolyte solution (°C), *L* the thickness of the specimen (mm), *x_d_* the average value of the penetration depths (mm), and *t* the test duration (hour).

#### 2.3.4. Chloride Content Test

To analyze the chloride content according to depth, NT Build 443 [31] and NT Build 208 [32] were implemented. The experiment aimed to analyze both chloride penetration depth and corresponding chloride content. Whereas the usual method for NT Build 443 requires specimens be immersed in NaCl solution for 35 days, the specimens were tested after 180 days in CaCl_2_ deicing solution. NT Build 208 analyzes chloride content by Volhard titration. With a similar methodology of sample preparation in Figure 1, chloride content was measured according to depth, up to 30 mm. The chloride content of specimens was calculated according to Equation (2):

(2) $$\%Cl^{-}=3.545\frac{V_{1}N_{1}-V_{2}N_{2}}{m},$$

where *V*_1_ is the added amount of silver nitrate solution (mL), *N*_1_ the normality of the silver nitrate solution, *V*_2_ the added amount of ammonium thiocyanate solution during titration (mL), *N*_2_ the normality of the ammonium thiocyanate solution, and *m* the weight of the sample (g).

#### 2.3.5. XRD Analysis

For the X-ray diffraction (XRD; Model SmartLab, Rigaku, Tokyo, Japan) analysis, an apparatus with CuK (Ni, filter), 35 kV, 20 mA, 2°/min scan speed, full scale, 140 cps, and 2θ = 5° to 60° specifications was used. The upper part of the specimens was cut into 10 mm thick discs horizontal to the principal axis. The first disc from the apex was discarded due to the direct contact with the solutions in which the specimens were exposed to. The second discs were used for XRD analysis. One millimeter of the samples was then ground from the lateral surface inward for XRD analysis to give results for different depths into the sample’s principal axis. The specimens used for XRD analysis were immersed in deicing solution for 180 days. Each specimen was analyzed five times at different depths of 1, 3, 5, 7, and 9 mm to investigate the hydration products as depth increases.

#### 2.3.6. SEM-EDAX Analysis

After the cylindrical specimens were immersed in deicing environments for 180 days, they were analyzed using scanning electron microscopy with energy dispersive X-ray spectroscopy (SEM-EDAX). In this study, a scanning electron microscope (Model S-3000 N, Hitachi, Tokyo, Japan) with 0.2–30 kV accelerating voltage, a 10 × 10^−12^~10 × 10^−5^ A probe current, a 3.5 mm secondary electron imaging (SEI) resolution (WD = 8 mm; Acc = 35 kV), and 10–300,000× magnifications was used to evaluate the microstructures. The samples were prepared as aforementioned in the XRD section. The 10-mm wide lateral surface was of interest and samples were taken from the disc as shown in Figure 1. Less than 1 mm from the lateral surface that was in direct contact with the solutions was removed in order to analyze the precise microstructure of the specimens that were affected by deicing environments.

## 3. Results and Discussion

### 3.1. Specimens Up to 28 Days

#### 3.1.1. Compressive Strength Test

**Figure 2:**
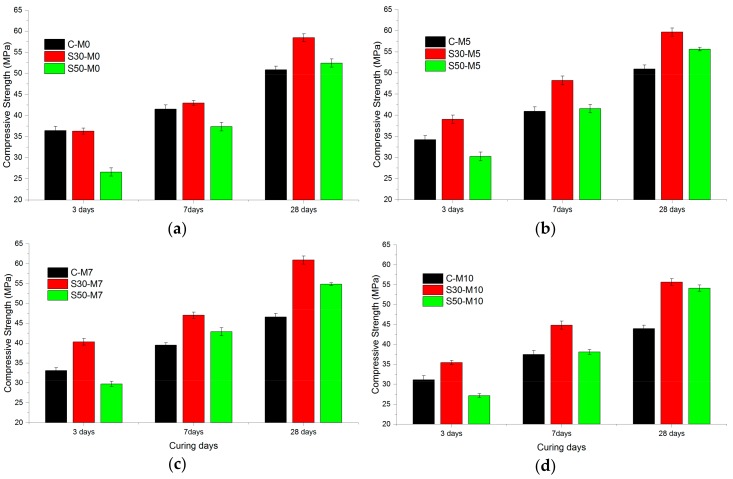
Compressive strength results of specimens at 3, 7 and 28 days, grouped according to MgO dosages of (**a**) 0; (**b**) 5%; (**c**) 7% and (**d**) 10%.

The compressive strength test results for specimens at 3, 7, and 28 days were measured and are shown in Figure 2. For each compressive strength value, three specimens were tested and the average value was used. In the C- specimens, the compressive strength of samples with MgO did not surpass those of the plain specimens, but C-M5 at 28 days surpassed C-M0 by a slight margin of 0.18%. The compressive strength at 28 days for C-M7 and C-M10 were lower than C-M0 by 8.38% and 13.52%, respectively. This could be attributed to the relatively slower hydration rate of MgO compared to the hydration rate of OPC [33]. The hydration of MgO additive is presented in Equation (3):

MgO + H_2_O → Mg^2+^ + 2OH^−^ → Mg(OH)_2_. (3)

Previous researches have shown that cement systems with MgO additives do not display greater compressive strength than those without in the short term [33]. It has also been reported that the hydration of MgO does not directly affect the strength growth of the cementing system when added to OPC cementing systems—the expansive characteristic of the formation of brucite has more to do with the shrinkage compensation [23].

In the S30- and S50- specimens, the specimens with MgO had higher compressive strength throughout the 28-day period compared to the ones without MgO, just for the exception of the case where the compressive strength of S30-M10 was lower than that of S30-M0 by 4.81%. S30-M7 showed the highest compressive strength in S30- samples with 60.89 MPa at 28 days. S50- specimens showed similar trends in compressive strength to those of S30- samples: compressive strength rose for specimens with MgO addition of 5% and more for 7% but showed slight decrease with 10%. The higher compressive strength of S30- and S50- specimens compared to C- specimens could be attributed to the formation of M-S-H phases in the cement matrix. In slag-blended cements, M-S-H products that are analogous with C-S-H have been reported to form [34]. Although M-S-H phases were once believed to have limited contribution to strength [35], recent studies have proposed that M-S-H gel has potential in developing strength in the cement matrix [21]. As is displayed in the results, using MgO in slag-blended cement increased compressive strength most notably between C-M7 and S30-M7 at 28 days, where the compressive strength of S30-M7 was higher by 30.81%. Studies have shown that the addition of MgO to existing C-S-H structures increases the pH values of the system up to 11.5 and will alter the Ca/Si ratio of the C-S-H structures from 0.8 to around 1.0 [36]. The strength test results from the S30- and S50- specimens are in accordance with past research regarding the formation of M-S-H in the tested specimens.

#### 3.1.2. Accelerated Carbonation

**Figure 3:**
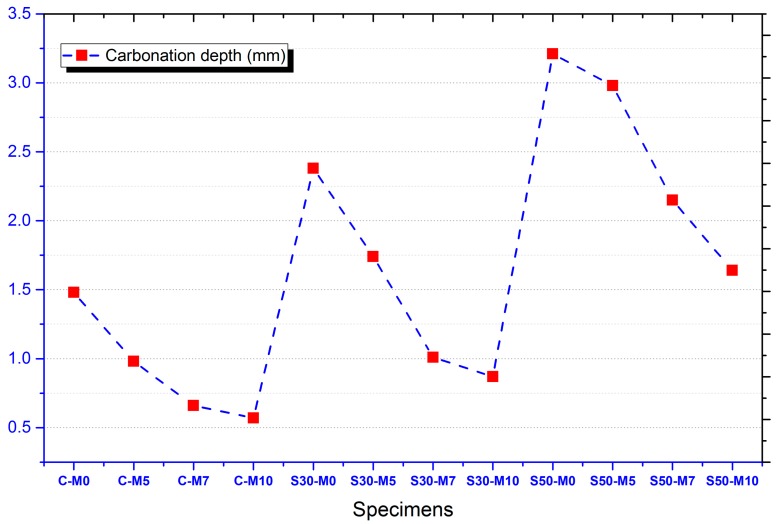
Carbonation depths of specimens.

The results of the accelerated carbonation test for specimens are presented in Figure 3. The carbonation depth was measured by using phenolphthalein pH indicator, with the specimen changing from colorless to pink when pH is higher than 9.0. The regions that remained colorless were measured to assess the carbonation depths. The most notable trend in C-, S30- and S50- specimens were the decrease in carbonation depth with increase in MgO content. This could be attributed to the formation of magnesite from MgO and carbon dioxide as presented in Equation (4), or the formation of nesquehonite as presented in Equation (5):

MgO + CO_2_ → MgCO_3_, (4)

MgO + CO_2_ + 3H_2_O → MgCO_3_∙3H_2_O. (5)

Previous studies have experimented on the accelerated carbonation of magnesia cements and have reported the formation of MgCO_3_∙3H_2_O and Mg(OH)_2_ with some samples reaching up to 20 MPa in compressive strength [37]. It can be inferred that like Ca(OH)_2_, Mg(OH)_2_ undergoes carbonation to form carbonate phases such as magnesite, nesquehonite, hydromagnesite (Mg_5_(CO_3_)_4_(OH)_2_∙4H_2_O), and dypingite (Mg_5_(CO_3_)_4_(OH)_2_∙5H_2_O) [38,39,40,41,42]. The formation of such phases could hinder the process of carbonation of concrete specimens and also densify the microstructure, which is in accordance with the experimental results of decreasing carbonation depth with increase in MgO content.

The relatively deeper carbonation depths of specimens with increased slag cement replacement levels could have been induced by the comparative lack of calcium content of the slag cement compared to OPC, inducing lower capacity for buffering the pH of the pore solution [43]. The specimens with slag cement also showed a decrease in carbonation depth with increase in MgO content. This is also in agreement with the carbonation results of the C- specimens, with MgO acting as a buffer for carbonation. The reactions between MgO and carbon dioxide near the surface could have acted as a means of protection for further progression of carbonation.

#### 3.1.3. Chloride Penetration Test

**Figure 4:**
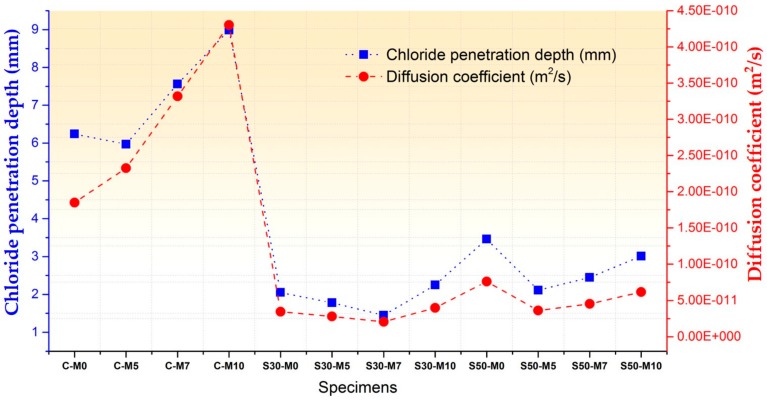
Chloride penetration depths and diffusion coefficients.

The durability characteristics of the specimens were evaluated by performing an accelerated chloride penetration test. Figure 4 presents the results of the chloride penetration test and corresponding diffusion coefficients. During the hydration phases, C- specimens showed higher diffusion coefficients with increase in MgO content. The relatively slower hydration rate of MgO in the C- specimens could have been a major factor in the specimens with MgO displaying higher diffusion coefficients, which is in accordance with the compressive strength test results. Slower hydration rate would mean that the cement matrix of systems with MgO are in some ways looser, with more interconnected pores in the cement matrix and cracks near the interfacial transition zone (ITZ).

In the S30- and S50- specimens, the diffusion coefficients are less in correspondence with the strength test results when compared with the C- specimens. Both sets of specimens with slag cement replacement, however, showed improved chloride penetration resistance when compared to C- specimens. The diffusion coefficient values are presented in Table 4. As was mentioned in Section 3.1.1., the additional C-S-H and M-S-H phases could have improved the cement matrix. Furthermore, the chloride binding capacity of additional C-S-H phases induced by the usage of slag cement has been reported to improve the resistance of cement systems to chloride penetrations [44]. As it can be seen in the lower diffusion coefficients of S30- and S50- specimens with MgO addition compared to S30-M0 and S50-M0 with the exception of S30-M10 specimen, the C-S-H and additional M-S-H phases could have been a factor in improving chloride penetration resistance. The analogous nature of M-S-H phases to C-S-H phases could provide an explanation for such results. As chloride penetration occurs from the surface in contact with solutions in the experimental setup, the diffusion paths in the cement matrix and ITZ could be more efficiently blocked by the various reactions between MgO and binders in the system. This would also be coherent with the compressive strength results.

#### 3.1.4. SEM-EDAX Analysis

**Figure 5:**
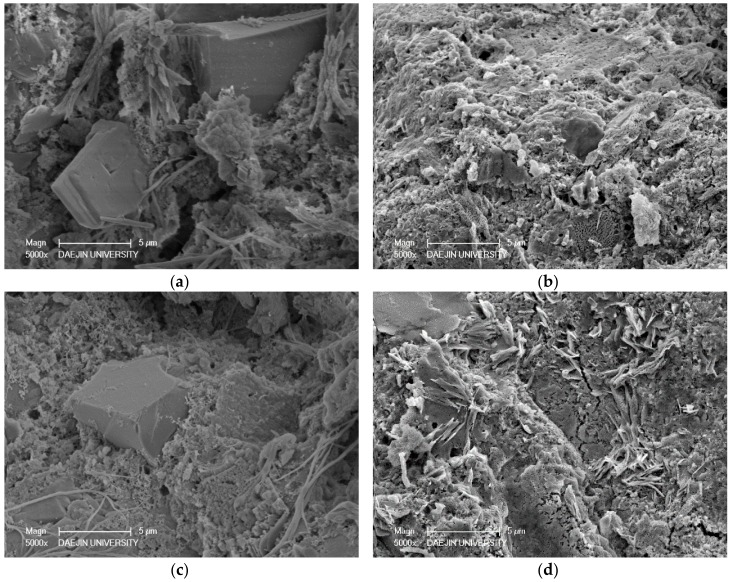
SEM-EDAX analysis of C-M0 and C-M5 specimens at 3 and 28 days: (**a**) C-M0 after 3 days; (**b**) C-M0 after 28 days; (**c**) C-M5 after 3 days; (**d**) C-M5 after 28 days.

**Figure 6:**
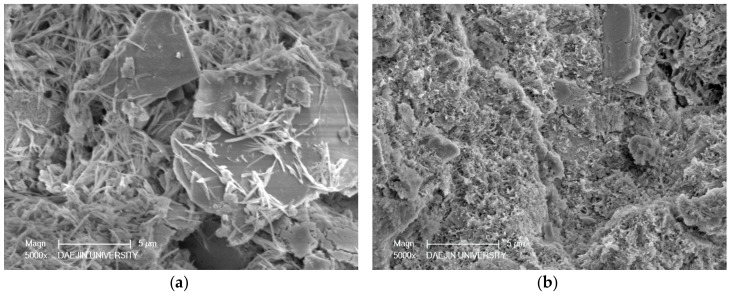

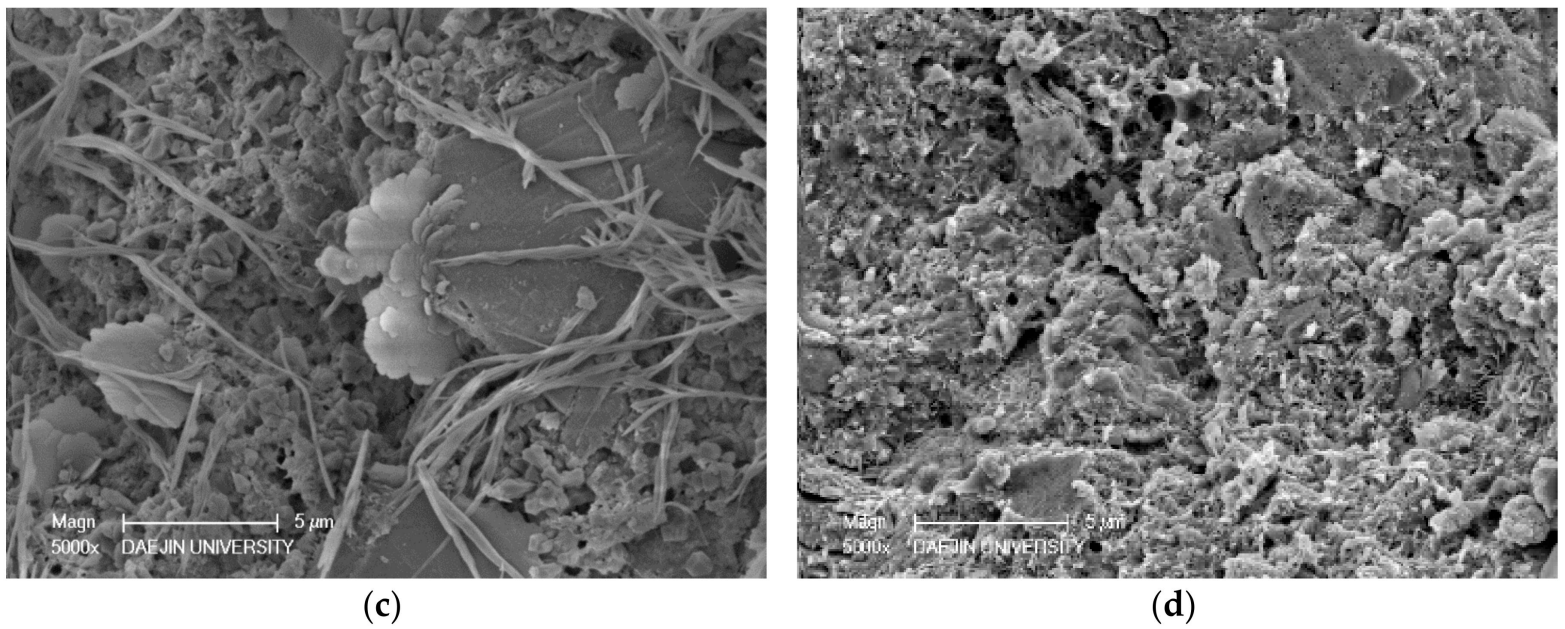
SEM-EDAX analysis of S30-M0 and S30-M5 specimens at 3 and 28 days: (**a**) S30-M0 after 3 days; (**b**) S30-M0 after 28 days; (**c**) S30-M5 after 3 days; (**d**) S30-M5 after 28 days.

**Figure 7:**
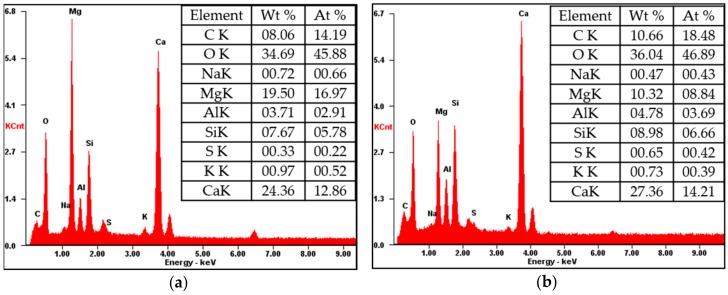
EDAX analysis of (**a**) C-M5; (**b**) S30-M5.

Two sets of specimens were chosen for SEM-EDAX analysis of hydration products: C- M0 and M5 and S30- M0 and M5. Each set compared the M0 and M5 specimens at 3 days and 28 days, and EDAX analysis was performed on the M5 samples at 28 days. The SEM analyses of C- M0 and M5 are presented in Figure 5, and the SEM analyses of S30- M0 and M5 are presented in Figure 6. The EDAX analyses of C-M5 and S30-M5 are presented in Figure 7.

In compliance with the compressive strength results, the degree of hydration at 3 days is greater in C-M0 than C-M5. As hydration progresses, the degree of hydration at 28 days is similar in C- M0 and M5, with the cement matrix showing similarly dense microstructures. The most notable difference is the products identified at 28 days. With the C-M5 specimen, the matrix is slightly denser after 28 days and MgO based products are visible. EDAX analysis shows an atomic ratio of approximately 17% of Mg, 13% of Ca, 6% of Si, and 46% of O, which could imply that brucite and CH were formed after 28 days and possibly some traces of C-S-H and M-S-H. The analyzed area could be a form of hydrated magnesium, with Mg showing to be highest without regarding O. Overall, CH, C-S-H, ettringite, M-S-H, and brucite could be identified from the specimens at 28 days.

From the SEM analyses for S30- M0 and M5, both specimens showed manifestations of CH and ettringite at 3 days. In compliance with the strength test results at 3 days, the microstructure of S30-M5 appears to be denser than S30-M0 and the degree of hydration greater. The amount of ettringite at 3 days is greater in S30-M0 compared to S30-M5, which further explains the more stable microstructure of S30-M5. At 28 days, the microstructure shows to be denser in S30-M5, and the hydration products show C-S-H and M-S-H phases. Comparing the S30- specimens to C- specimens, the manifestation of C-S-H and M-S-H are more visible. This could explain the increased compressive strength in the S30- specimens and could also imply that the usage of slag cement and MgO can improve the cement matrix. Further EDAX analysis on the S30-M5 specimen shows an atomic ratio of approximately 9% of Mg, 7% of Si, 14% of Ca, and 47% of O. It could be inferred from the results that the analyzed area consists of M-S-H and C-S-H structures.

### 3.2. Specimens after Immersion in Deicer Solution Up to 180 Days

#### 3.2.1. Compressive Strength

**Figure 8:**
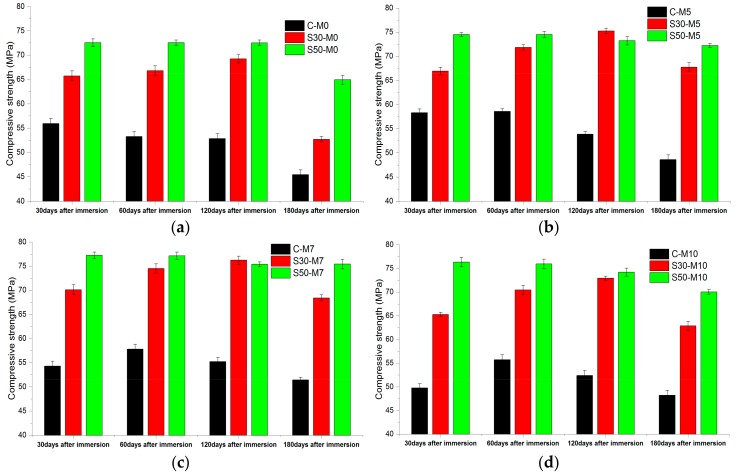
Compressive strength results of specimens immersed in CaCl_2_ deicing solution for 30, 60, 120 and 180 days, grouped according to MgO dosages of (**a**) 0; (**b**) 5%; (**c**) 7% and (**d**) 10%.

To analyze the strength development and deterioration of specimens with different binders and MgO dosages, compressive strength tests were conducted at different stages of the immersion period. The compressive strengths of the specimens immersed in CaCl_2_ deicing solution for 30, 60, 120, and 180 days are shown in Figure 8. All of the C- specimens showed increases in compressive strength during the first 30 days in the deicing solution: C- M0, M5, M7, and M10 increased by 10.08%, 14.58%, 16.61%, and 13.25% respectively. The C-M0 specimens then lost strength at 60, 120, and 180 days by 4.75%, 0.86%, and 13.96% compared to the preceding specimens. The C- M5, M7, and M10 samples, however, gained strength until 60 days of immersion by 0.43%, 6.52%, and 12.02%, respectively, compared to the compressive strength of each samples at 30 days of immersion. The specimens with MgO gaining strength for longer a period of time compared to the specimen without MgO could be attributed to the formation of brucite prior to being exposed to the deleterious effects of CaCl_2_ deicing solution. It has been reported that the usage of MgO refines the microstructure of the cement matrix by expanding locally, thus causing expansion to compensate autogenous shrinkage and thermal shrinkage [45]. The higher compressive strength under extreme conditions (here as in 30% CaCl_2_ by mass) could be explained by such refinement of the cement matrix microstructure.

S30- specimens have shown strength gain up to 120 days in immersion. The compressive strength values at 120 days in deicer solution in comparison to those of 28 days of water-curing increased by 18.43%, 26.17%, 25.21%, and 31.12% for S30- M0, M5, M7, and M10, respectively. The strength values of S30-M7 samples were higher than other S30- samples, 10.13% higher than the S30-M0 specimen at 120 days in solution. After 180 days in the deicing solution, the S30- M0, M5, M7, and M10 lost compressive strength by 23.91%, 9.98%, 10.26%, and 13.79%, respectively, compared to the compressive strength at 120 days. After 180 days, the compressive strength of S50- specimens were the highest from the specimens with the same MgO addition. The compressive strength values of S50- M0, M5, M7, and M10 were higher by 42.87%, 48.51%, 46.71%, and 45.28% than corresponding C- specimens, and 23.23%, 6.60%, 10.26%, and 11.40% higher than corresponding S30- specimens. S30- and S50- specimens gaining strength for longer periods of time in the solution compared to the C- specimens could be attributed to the combined effect of MgO hydration and the additional C-S-H phases that the slag replacement has induced. The chloride binding capacity of the C-S-H phase and the refinement of the cement matrix with slag cement could have enhanced the specimens’ durability in the deicer solution [46]. The decalcification of C-S-H and the subsequent formation of M-S-H has been reported in maritime concrete structures and in concrete systems exposed to the deicing environment [47]. The presence of M-S-H structures could have prevented the damage from the decalcification of C-S-H structures, resulting in less damages from deicers.

#### 3.2.2. Chloride Content According to Depth

**Figure 9:**
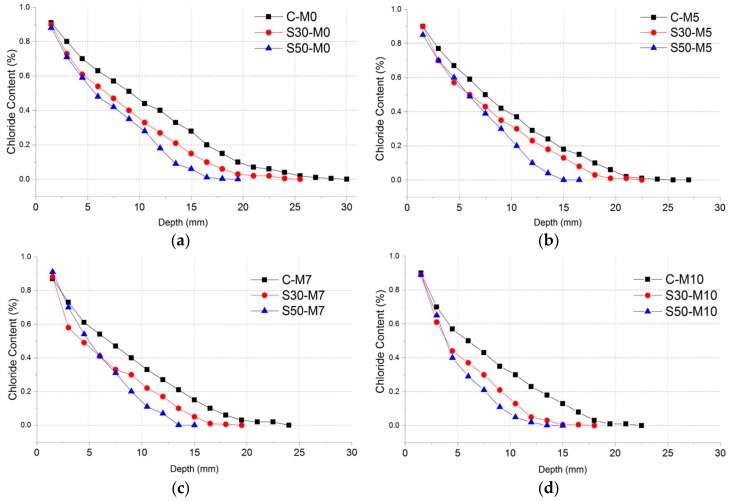
Chloride content profile of specimens after 180 days of immersion, grouped according to MgO dosages of (**a**) 0; (**b**) 5%; (**c**) 7% and (**d**) 10%.

To analyze the depth of chloride ingress and chloride profile after 180 days of immersion in the deicing solution, Volhard titration was conducted according to depth. The results of the chloride content test are shown in Figure 9. In the C- specimens, the increase in MgO addition resulted in a decrease of chloride penetration depth. The C-M0 specimen showed approximately 29 mm of penetration depth, while the C-M10 specimen showed 22 mm of penetration depth. Such trends were also found in S30- and S50- specimens. S30-M0 showed penetration depth of 25 mm and S30-M10 showed 18 mm. S50-M0 specimen showed 18 mm of depth and S50-M10 showed the lowest penetration depth of all specimens with 15 mm.

The similar trends shown in the specimens after immersion could imply that after the hydration phases, the addition of MgO to the cement matrix improved chloride penetration resistance. In the C- specimens, the maximum depth where chloride content could be identified decreased by 24.14% compared C- M10 to M0. This could infer that the addition of MgO and the subsequent manifestation of magnesium-based hydration products could enhance the cement matrix in the long term in conditions with external chloride. In Section 3.1.3., the relatively slower hydration rate of MgO resulted in reduced chloride penetration resistance in specimens with MgO. Here, however, after 28 days of hydration and subsequent immersion in deicing solution for 180 days, specimens with MgO showed improved chloride penetration resistance. The expansive characteristics of MgO and its hydration products could have affected the microstructure and made it denser. Furthermore, the chemical damages induced by the CaCl_2_ solution could have been mitigated by the addition of MgO and its hydration products. The S30- and S50- specimens showed improvement compared to the C- specimens and showed similar trends of the penetration depth decreasing with the increase in MgO addition, and S50- showed improved chloride resistance from S30- specimens.

#### 3.2.3. XRD Analysis According to Depth from the Surface to the Inside

**Figure 10:**
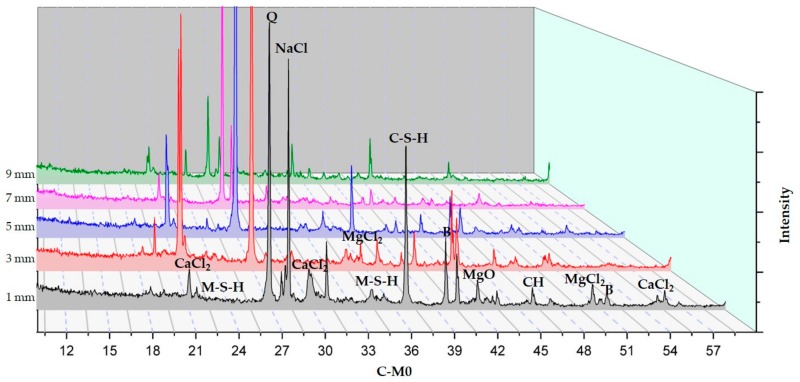
XRD results of C-M0.

**Figure 11:**
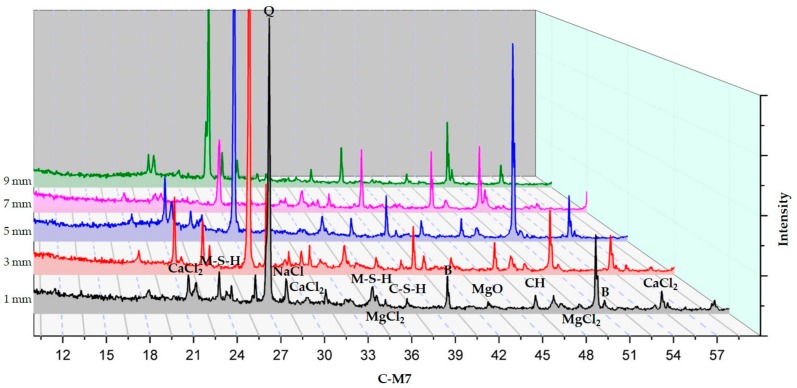
XRD results of C-M7.

**Figure 12:**
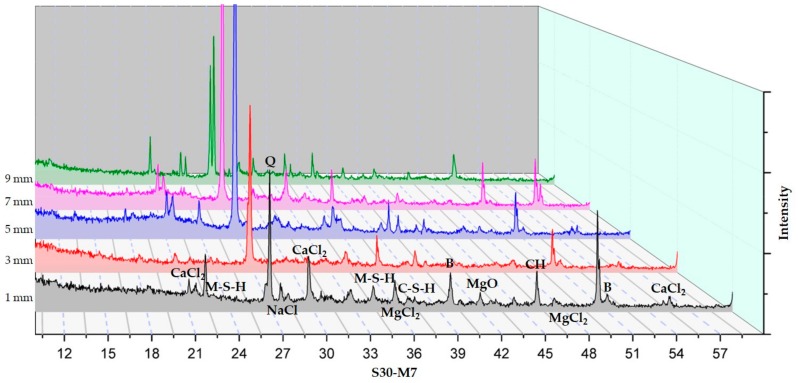
XRD results of S30-M7.

The XRD analysis according to depth of C-M0, C-M7, and S30-M7 are presented in Figure 10, Figure 11 and Figure 12 respectively.

XRD analyses of the specimens indicated the presence of brucite, M-S-H, CH, C-S-H, CaCl_2_, MgCl_2_, NaCl, and MgO. In comparing the XRD graphs for C-M0 and C-M7, the most notable difference is the manifestation of brucite at around 40° and 51.5°. C-M0 shows an intense peak at 40° and it gradually decreases as the depth increases. C-M7 sample shows a different trend in brucite formation, where the peak gradually increases. Similar trends can be seen with M-S-H phases at 34.5° and 23.5°. The formation of M-S-H is mostly near the surface in the C-M0 specimen, while the peak is higher and more evenly distributed in the C-M7 specimens. This could be an implication that brucite and M-S-H formation from the added MgO were stable throughout the sample and had beneficial effects on the cement matrix as was shown in the compressive strength test results. Lightly burnt MgO hydrates locally, and this could explain the relatively even distribution of brucite in the sample. In the C-M0 specimens, brucite and M-S-H formation is mainly concentrated near the surface. This could be due to the effects of the CaCl_2_ deicer solution leaching CH and decalcifying C-S-H at the surface and inducing brucite formation at the surface [2]. Furthermore, the peak intensities of MgCl_2_ are lower for C-M7 specimens than C-M0 specimens. This could also be attributed to the more stable hydration of the lightly-burnt MgO additive. The presiding dead burnt MgO in cement clinkers have a slower hydration rate than lightly burnt MgO additive in the C-M7 specimens [14]. The CaCl_2_ deicing solution could have caused the MgCl_2_ peaks at 50° and 35° to be higher in the C-M0 specimens. Furthermore, the CaCl_2_ peak in the C-M0 specimen around 21°, 30°, and 55.5° are higher than the C-M7 specimen. This could imply that the specimens with MgO are less susceptible to leaching of CH when compared to the specimens without MgO addition.

In comparing the S30-M7 to C-M7 specimens, the peak intensities of CaCl_2_ around 21°, 30°, and 55.5° for the S30-M7 specimens are generally lower throughout the specimens’ depth, except for the peak nearest to the surface around 30°. Also, the peak intensities for CH and C-S-H are higher for S30-M7 than C-M7. This could be an indication of the S30-M7 specimens having improved resistance to the deicer solution, thus having CH and C-S-H phases that are more intact. Furthermore, the peak intensities for M-S-H are relatively evenly distributed for different depths. In S30-M7, the peaks for CaCl_2_ generally decline with depth, which can be associated with improved resistance to chloride attack. The lower peak intensities of MgCl_2_, CaCl_2_, and NaCl compared to the two C- specimens show corresponding results with the compressive strength and chloride content results. The lower peak intensities of chloride-based compounds could be explained by the additional manifestation of M-S-H and C-S-H phases the usage of slag cement and MgO induces. This could in turn be a beneficial factor in increasing chloride attack resistance and chemical damage resistance.

#### 3.2.4. SEM-EDAX Analysis

**Figure 13:**
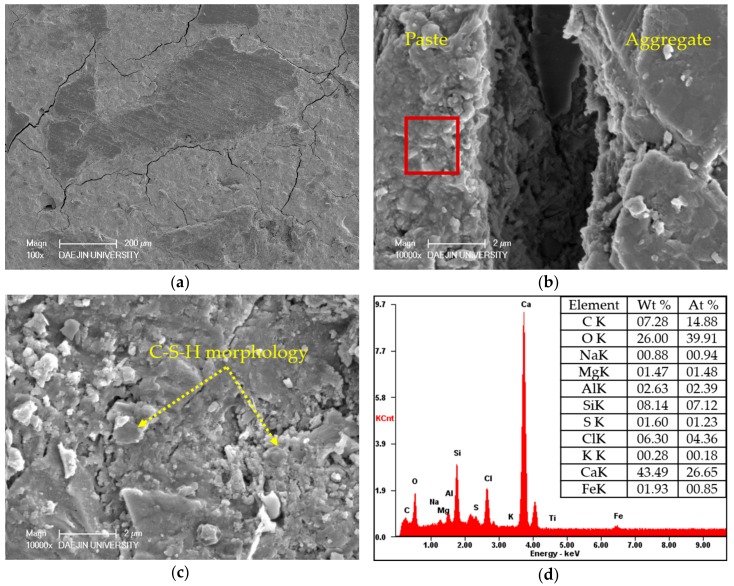
SEM-EDAX analysis of C-M0 after 180 days of immersion in deicing solution: (**a**) crack formation; (**b**) ITZ; (**c**) observation of hydration products; (**d**) EDAX analysis of the area enclosed by the red square in (**b**).

**Figure 14:**
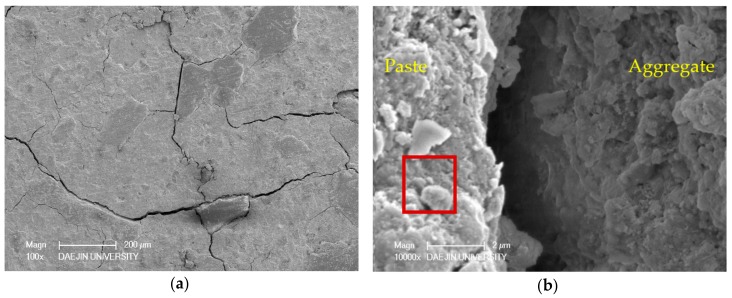

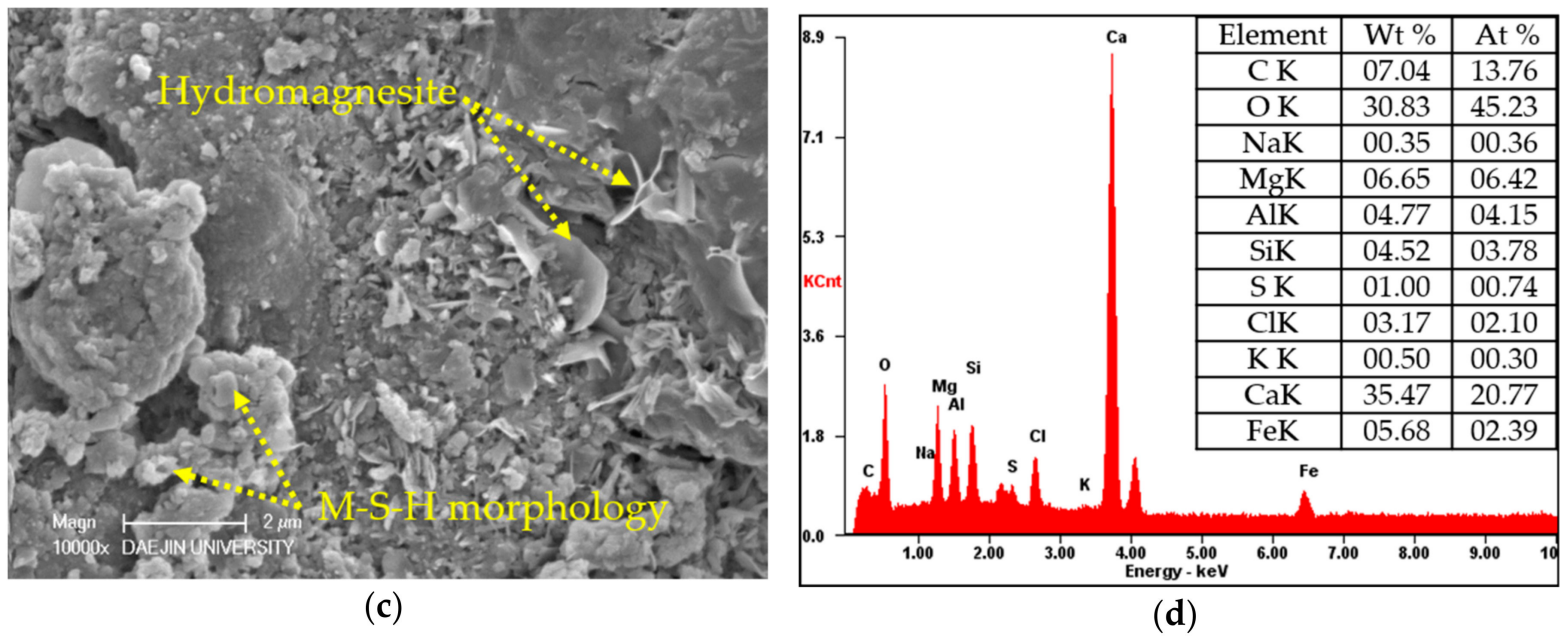
SEM-EDAX analysis of C-M5 after 180 days of immersion in deicing solution: (**a**) crack formation; (**b**) ITZ; (**c**) observation of hydration products; (**d**) EDAX analysis of the area enclosed by the red square in (**b**).

**Figure 15:**
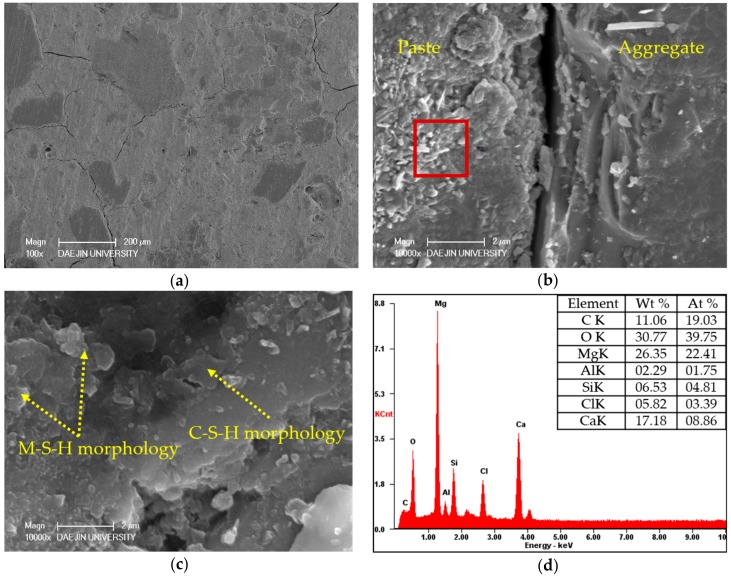
SEM-EDAX analysis of S30-M7 after 180 days of immersion in deicing solution: (**a**) crack formation; (**b**) ITZ; (**c**) observation of hydration products; (**d**) EDAX analysis of the area enclosed by the red square in (**b**).

The SEM-EDAX analysis of C-M0, C-M5, and S30-M7 are presented in Figure 13, Figure 14 and Figure 15, respectively. The specimens were immersed in deicing solution for 180 days prior to the analysis and the crack formations are examined at magnification of ×100, the ITZ and cement matrix at ×10,000.

From the ×100 SEM images of the specimens, S30-M7 showed the least crack formation and the specimen was very much intact. The cracks on the C- specimens were larger in size and number compared to the S30-M7 specimen. Also, the ITZ of S30-M7 is narrower and more intact. From the ×10,000 SEM images, the hydration products of C-M0 specimen shows the formation of C-S-H phases, and the C-M5 specimen shows manifestations of M-S-H morphology and hydromagnesite. The addition of MgO in the C-M5 specimen could have induced the formation of M-S-H phases and hydrated magnesium carbonates. The EDAX results also show a higher content of Mg in the C-M5 specimen compared to the C-M0 specimen, with an atomic ratio of 6.42%. C-M5 also shows traces of Si and Cl, which could be an indication of the M-S-H morphology identified in the SEM image and perhaps bound chloride ions. The specimens were immersed in deicing solution, which would explain the presence of chloride ions. The formation of hydromagnesite or other hydrated magnesium carbonates in specimens with MgO are similar in their nature with hydrated calcium carbonates—MgO hydrates and becomes brucite, then reacts with carbon dioxide. From the SEM image of S30-M7 specimen, M-S-H phases and C-S-H phases are identified. The addition of slag cement to concrete systems with MgO addition has been reported to form M-S-H gel phases, and that C-S-H gel phases and M-S-H gel phases coexist [34], which the SEM images confirm. The EDAX results show presence of Mg at approximately 22% by atomic weight, with traces of Ca and Si at approximately 9% and 5%, respectively. 

## 4. Conclusions

Building on past researches that have shown the mechanisms of calcium chloride damages, extensive tests on the mechanical properties and durability of concrete systems with MgO and slag cement showed the following conclusions.

As can be seen from the compressive strength results at 28 days after water-curing, MgO hydrates at a slower rate compared to OPC; however, in systems with slag cement, MgO manifests as M-S-H phases with C-S-H phases. The carbonation results show an increase in resistance with addition of MgO due to the magnesium carbonate phases that block the diffusion path. Furthermore, from the chloride penetration results, it can be verified that the addition of MgO to OPC systems decreases the chloride penetration resistance. However, in systems with slag cement, chloride penetration resistance is improved with the addition of MgO. This also could be caused by the additional M-S-H phases that could improve chloride binding capacity and also improve the microstructure. The SEM-EDAX results show the hydration process up to 28 days, which are in accordance with the preceding experiments.

After the immersion of specimens in the CaCl_2_ deicing solution, the damages caused by deicers can be evaluated from the compressive strength tests. C-M0 specimens deteriorated earlier than C- specimens with MgO, showing 13.96% lesser compressive strength at 180 days compared to the compressive strength at 30 days. S30- and S50- specimens showed greater resistance to deicers, with S30-M7 and S50-M7 showing the highest compressive strengths at 120 days and 180 days after immersion. S50- specimens displayed the highest compressive strength values after 180 days in comparison to other specimens with the same amount of MgO added. The chloride content test also supports the compressive strength values, with the penetration depths decreasing in similar fashion to the increases in compressive strengths. The addition of MgO and replacement by slag cement greatly improves the concrete systems’ resistance to deicers and chloride penetration. This could be due to the combined effects of already well documented benefits of slag cement and the effects of MgO when added to systems with slag cement. Additional C-S-H phase and M-S-H phases could all increase chloride binding capacity and refined the microstructure of the cement matrices.

The XRD analysis according to depth provides a profile of the specimens exposed to deicers up to 9 mm. The XRD results indicate the presence of brucite, M-S-H, CH, C-S-H, CaCl_2_, MgCl_2_, NaCl, and MgO. Comparing C-M0 and C-M7, the addition of MgO could have induced the more even distribution of MgO based compounds, and the peak intensities of MgCl_2_ are lower in C-M7. The stability of and the local hydration of MgO in the C-M7 could have improved the concrete system’s stability in CaCl_2_ conditions. S30-M7 specimen shows higher peaks for C-S-H phases and M-S-H phases. Furthermore, chloride-based salts are less prevalent throughout the specimen compared to the C- specimens. From the SEM-EDAX analysis, crack formations show the relative stability of specimens with MgO, and the ITZ is more intact. Magnesium carbonates can be found on the C-M5 specimen, and the coexistence of C-S-H phases and M-S-H phases can be found on the S30-M7 specimen. These indications from the hydration products are in accordance with the premises that MgO addition with slag cement replacement results in the formation of M-S-H phases, thus making the concrete system more durable in deicing conditions. The conclusions drawn above indicate that the usage of slag cement and MgO could be an effective method of mitigating chemical and mechanical damages in pavement systems. The combined effects of slag cement and MgO have shown manifestations of C-S-H and M-S-H phases and have been shown to effectively mitigate chloride penetration. After 180 days of immersion in 30% CaCl_2_ solution, the system with slag cement and MgO withstood the damages much more effectively. Although the system still needs further experiments regarding physical and mechanical damages, the results of this study deem the system to be a possible alternative to pavement systems in use.

## Figures and Tables

**Table 1:** Chemical composition and physical properties of binders.

Binder	SiO_2_ (%)	Al_2_O_3_ (%)	Fe_2_O_3_ (%)	CaO (%)	MgO (%)	SO_3_ (%)	Ig. loss (%)	Specific Gravity	Surface Area (cm^2^/g)
OPC	20.8	6.3	3.2	62.0	3.3	2.2	1.5	3.15	3410
Slag cement	34.1	16.1	0.4	42.3	4.1	2.5	0.05	2.89	4893
MgO	3.34	0.54	2.18	4.14	88.5	0.04	-	-	-

**Table 2:** Physical properties of the aggregates.

Properties	Fine Aggregate	Coarse Aggregate	Test Method
G_max_ (mm)	25	25	-
Density (g/cm^3^)	2.62	2.66	KS F 2503
Bulk absorption (%)	0.72	0.72	KS F 2503
Fineness modulus	6.91	6.91	KS F 2502
Abrasion rate (%)	25.1	25.1	KS F 2508
Unit volume mass (kg/L)	1.564	1.564	KS F 2505

**Table 3:** Mix proportion of concrete specimens.

Types	W/B (%)	S/a (%)	Unit Weight (kg/m^3^)						
			Water	OPC	Slag Cement	Sand	Gravel	MgO	SP
C-M0	42.5	39.2	184	499	-	618	965	-	4.99 (C×1%)
C-M5								24.95	
C-M7								34.93	
C-M10								49.9	
S30-M0				349.3	149.7			-	
S30-M5								24.95	
S30-M7								34.93	
S30-M10								49.9	
S50-M0				249.5	249.5			-	
S50-M5								24.95	
S50-M7								34.93	
S50-M10								49.9	

**Table 4:** Diffusion coefficient values of all specimens (m^2^/s).

Binder Type	M0	M5	M7	M10
C-	1.85 × 10^−10^	2.33 × 10^−10^	3.32 × 10^−10^	4.30 × 10^−10^
S30-	3.46 × 10^−11^	2.79 × 10^−11^	2.05 × 10^−11^	3.98 × 10^−11^
S50-	7.62 × 10^−11^	3.61 × 10^−11^	4.53 × 10^−11^	6.17 × 10^−11^
